# Transperitoneal administration of dissolved hydrogen for peritoneal dialysis patients: a novel approach to suppress oxidative stress in the peritoneal cavity

**DOI:** 10.1186/2045-9912-3-14

**Published:** 2013-07-01

**Authors:** Hiroyuki Terawaki, Yoshimitsu Hayashi, Wan-Jun Zhu, Yukie Matsuyama, Tomoyoshi Terada, Shigeru Kabayama, Tsuyoshi Watanabe, Seiichi Era, Bunpei Sato, Masaaki Nakayama

**Affiliations:** 1 Dialysis Center, Fukushima Medical University, Fukushima, Japan; 2 Department of Nephrology, Hypertension, Diabetes, Endocrinology, and Metabolism, Fukushima Medical University, Fukushima, Japan; 3 Center for Advanced and Integrated Renal Science, Tohoku University Graduate School of Medicine, Sendai, Japan; 4 Department of Physiology and Biophysics, Gifu University Graduate School of Medicine, Gifu, Japan; 5 MiZ Company, Kanagawa, Japan

**Keywords:** Molecular hydrogen, Oxidative stress, Albumin redox state, Peritoneal dialysis

## Abstract

**Background:**

Oxidative stress (OS) related to glucose degradation products such as methylglyoxal is reportedly associated with peritoneal deterioration in patients treated with peritoneal dialysis (PD). However, the use of general antioxidant agents is limited due to their harmful effects. This study aimed to clarify the influence of the novel antioxidant molecular hydrogen (H_2_) on peritoneal OS using albumin redox state as a marker.

**Methods:**

Effluent and blood samples of 6 regular PD patients were obtained during the peritoneal equilibrium test using standard dialysate and hydrogen-enriched dialysate. The redox state of albumin in effluent and blood was determined using high-performance liquid chromatography.

**Results:**

Mean proportion of reduced albumin (ƒ(HMA)) in effluent was significantly higher in H_2_-enriched dialysate (62.31 ± 11.10%) than in standard dialysate (54.70 ± 13.08%). Likewise, serum ƒ(HMA) after administration of hydrogen-enriched dialysate (65.75 ± 7.52%) was significantly higher than that after standard dialysate (62.44 ± 7.66%).

**Conclusions:**

Trans-peritoneal administration of H_2_ reduces peritoneal and systemic OS.

## Background

Peritoneal deterioration is one of the most serious complications of peritoneal dialysis (PD) therapy, leading to ultrafiltration failure and the more severe complication of encapsulating peritoneal sclerosis (EPS). As the duration of PD increases, so does the risk of peritoneal deterioration [1]. More than 40% of patients in Japan who were on PD treatment for longer than 8 years stopped it due to the progression of peritoneal damage [2]. The pathological mechanisms of peritoneal damage are multi-factorial, but accumulated data have revealed the critical role of glucose degradation end-products (GDPs), i.e., chemically reactive carbonyl compounds. Methylglyoxal (MG) is one of the representative toxic GDPs, causing detrimental effects due to its rapid and indiscriminate oxidative nature [3], and its production of toxic reactive oxygen species (ROS) such as hydroxyl radical, methyl radical, and undetermined carbon-centered radicals [4]. These used to be present in conventional dialysate, and also enter into the dialysate from uremic plasma [5]. Bio-compatible low-GDP dialysate is currently available, but a Japanese multicenter nationwide study, the NEXT-PD study [6], revealed the occurrence of EPS even with the use of low-GDP solutions [under submission]. This indicates the need for novel therapeutic approaches to suppress possible insults from enhanced oxidative stress (OS) due to uremic oxidants in the peritoneal cavity.

Recently, the novel role of molecular hydrogen (H_2_) as an antioxidant has been revealed. H_2_ eliminates the hydroxyl radical in cultured cells and living organisms [7]. Interestingly, H_2_ does not influence other ROS, including superoxide, peroxide, and nitric oxide; these ROS play important physiological roles in body [8]. In humans, the safety of H_2_ has been tested, particularly in the field of deep diving. In contrast to general drugs, which usually have some harmful effects, no toxicity was found even at high concentrations of H_2_[9]. H_2_ thus has therapeutic potential for pathological states related to ROS [10].

The present study tested the effects of peritoneal dialysate containing a high concentration of molecular hydrogen (H_2_-enriched dialysate) as a novel anti-oxidant among patients treated with PD. As a result, we demonstrated that the use of hydrogen-enriched dialysate could reduce not only peritoneal, but also systemic OS in clinical settings.

## Methods

### Preparation of hydrogen-enriched dialysate

**Figure 1:**
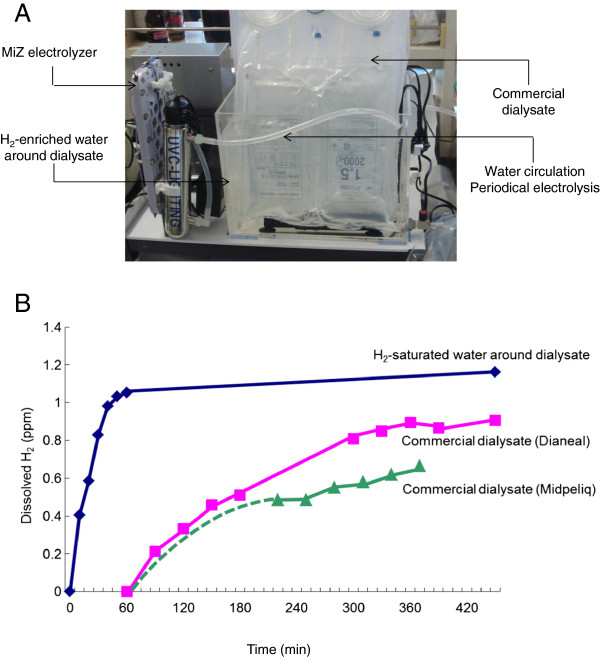
**MiZ nondestructive hydrogen dissolver (A) and the hydrogen concentration of peritoneal dialysate in hydrogen-saturated water (B).** Hydrogen concentration of dialysate and hydrogen-saturated water around dialysate was measured using a dissolved H_2_ measurement apparatus DH-35A (DKK-TOA, Tokyo, Japan).

Hydrogen-enriched dialysate was prepared using MiZ nondestructive hydrogen dissolver (MiZ, Kanagawa, Japan), as reported elsewhere [11]. When commercial peritoneal dialysate is immersed in H_2_-enriched water, hydrogen permeates through the container, resulting in the H_2_ concentration of dialysate gradually increasing in a time-dependent manner (Figure 1). We prepared H_2_-enriched dialysate using this apparatus by immersing commercial peritoneal dialysate bags for more than 2 hr. Hydrogen-enriched dialysate was then applied as a test solution for peritoneal equilibrium testing.

### Patients

Six male PD patients were studied (mean age, 55 years; range, 44–71 years; length of PD, 39 ± 17 months; weight, 68.1 ± 16.1 kg; height, 166.2 ± 5.6 cm). The pathology underlying end-stage renal disease was as follows: chronic glomerulonephritis, n = 3; diabetic nephropathy, n = 2; and hypertensive nephropathy, n = 1. Patients with active infection, bleeding, liver dysfunction, collagen disease, systemic vasculitis, cardiovascular accident within 6 months, or malignancy were excluded from this study. Performance status of all patients was class 1 according to American Heart Association criteria [12]. All patients had been receiving daily continuous ambulatory PD (3–4 bags/day) using neutral low-GDP dextrose solution. The ethics committee of Fukushima Medical University approved this study protocol (Acceptance No. 1362) and written informed consent was obtained from all patients prior to enrollment.

### Protocol

Patients underwent a simplified peritoneal equilibration test (fast PET) using standard dialysate, then underwent fast PET using hydrogen-enriched dialysate 2 weeks later. Fast PET was conducted in accordance with the method of Twardowski [13]. In brief, peritoneal dialysate (2 L of 2.5% dextrose-dialysate) was intraperitoneally infused with a Tenckhoff catheter, and the entire volume of dialysate was drained from the body after 240 min. The drained effluent was mixed well and 2 mL was collected as an effluent sample. Blood samples were obtained before and after fast PET, then 2 mL of serum was drawn after centrifugation and stored at −80°C for 1–4 weeks until analysis. Samples of serum and effluent collected to measure albumin redox were stored at −80°C for 1–4 weeks until analysis. During fast PET, blood pressure, cardiac pulse, and hydrogen concentration in the breath were measured repeatedly every 60 min. Breath hydrogen concentration was also measured in three cases just after, 15 min after, and 30 min after infusion of H_2_-enriched dialysate. Breath hydrogen concentration was measured using a biological gas (gas in the oral cavity) H_2_ measurement apparatus BGA-1000D (Aptec, Kyoto, Japan).

### Measurement of albumin redox state

Human serum albumin (HSA) is a protein composed of 585 amino acids. The amino residue at position 34 from the N-terminus is a cysteine, containing a mercapto group (SH group). This mercapto group deoxidizes other substances according to the degree of surrounding OS and is itself oxidized. From the perspective of cysteine residues, HSA is a mixture of human mercaptoalbumin (HMA) in which the mercapto group is not oxidized, human non-mercaptoalbumin-1 in which disulfide bond formation is reversibly oxidized mainly by cysteine (HNA-1), and human non-mercaptoalbumin-2 which is strongly oxidized and forms a sulfinic (−SO_2_H) or sulfonic (−SO_3_H) group.

The redox state of HSA was determined using high-performance liquid chromatography (HPLC), as previously reported [14]. The HPLC system consisted of an autosampler (AS-8010; Tosoh, Tokyo, Japan; injection volume, 2 μL) and double-plunger pump (CCPM; Tosoh) in conjunction with a system controller (CO-8011; Tosoh). Chromatographs were obtained using a UV6000LP photodiode alley detector (detection area, 200–600 nm with 1-nm step; Thermo Electron, Waltham, MA, USA). A Shodex-Asahipak ES-502N 7C column (10 × 0.76 cm I.D., DEAE-form for ion-exchange HPLC; Showa Denko, Tokyo, Japan; column temperature, 35 ± 0.5°C) was used in this study. Elusion was performed as linear gradient elusion with graded ethanol concentrations (0 to 1 min, 0%; 1 to 50 min, 0 → 10%; 50 to 55 min, 10 → 0%; 55 to 60 min, 0%) for serum in 0.05 M sodium acetate and 0.40 M sodium sulfate mixture (pH 4.85) at a flow rate of 1.0 mL/min. De-aeration of the buffer solution was performed by bubbling helium.

HPLC profiles obtained from these procedures were subjected to numerical curve fitting with PeakFit version 4.05 simulation software (SPSS Science, Chicago, IL, USA), and each peak shape was approximated by a Gaussian function. Values for fractions of HMA, HNA-1, and HNA-2 to total HSA were then calculated (ƒ(HMA), ƒ(HNA-1), and ƒ(HNA-2), respectively).

### Statistical analysis

Values are expressed as mean ± standard deviation unless otherwise stated. StatView version 5.0 statistical software (SAS Institute, Cary, NC, USA) was used for statistical analysis. The significance of collected data was evaluated using a paired *t*-test or 1-factor repeated-measures analysis of variance (ANOVA) followed by Scheffe’s test as a post-hoc test, as appropriate. For magnitude of correlation, Pearson’s correlation coefficient (*R*) was used. Differences or correlations were considered significant for values of *P* < 0.05.

## Results

Table 1 shows changes in blood pressure, heart rate, and breath hydrogen concentration during fast PET. Regarding blood pressure and heart rate, no significant difference was seen between standard and H_2_-enriched dialysate (paired *t*-test). No significant changes were observed during fast PET in either standard or H_2_-enriched dialysate (1-factor repeated-measures ANOVA).

**Table 1:** The changes of blood pressure, cardiac pulse, and breath H2 concentration during fast PET

	**Standard dialysate**	**H2-enriched dialysate**
Blood pressure mmHg		
0 min	130 ± 12 / 79 ± 10	135 ± 13 / 81 ± 10
60 min	130 ± 11 / 79 ± 5	131 ± 14 / 82 ± 12
120 min	125 ± 9 / 79 ± 7	134 ± 8 / 80 ± 14
180 min	123 ± 12 / 75 ± 12	136 ± 5 / 78 ± 12
240 min	128 ± 9 / 78 ± 7	132 ± 9 / 81 ± 13
Pulse /min		
0 min	81 ± 7	82 ± 12
60 min	76 ± 6	79 ± 12
120 min	74 ± 6	78 ± 14
180 min	77 ± 4	78 ± 17
240 min	78 ± 7	81 ± 15
Breath H2 ppm		
0 min	4.7 ± 6.6	3.2 ± 2.0
60 min	1.8 ± 1.3	8.3 ± 7.5*
120 min	3.0 ± 1.7	8.5 ± 11.0
180 min	4.2 ± 2.8	5.8 ± 4.8
240 min	5.5 ± 6.7	7.2 ± 4.6

*; p < 0.05 vs. standard dialysate.

**Figure 2:**
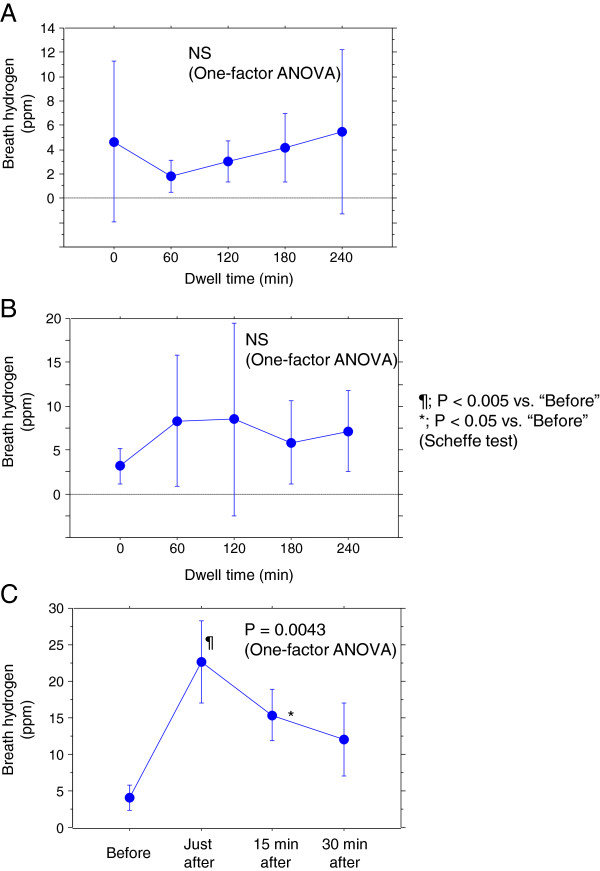
**Change in breath hydrogen concentration during fast PET. A**) Hourly change in PET using standard dialysate. No significant changes were observed. **B**) Hourly change during PET using H_2_-enriched dialysate. The hydrogen concentration at 60 min was significantly higher in H_2_-enriched dialysate than in standard dialysate. **C**) Breath hydrogen concentrations before, just after, 15 min after, and 30 min after administration of H_2_-enriched dialysate in three cases. Hydrogen concentrations just after and 15 min after administration were significantly higher than that before administration.

Changes in breath hydrogen concentration in all cases are shown in Table 1 and Figure 2 (A, B). Although no significant changes were observed during fast PET in both standard and H_2_-enriched dialysate, the hydrogen concentration at 60 min was significantly higher in H_2_-enriched dialysate than in standard dialysate.

Breath hydrogen concentrations before, just after, 15 min after, and 30 min after administration of H_2_-enriched dialysate in three cases are shown in Figure 2C. Hydrogen concentrations were significantly higher just after and 15 min after administration (22.7 ± 5.7 and 15.3 ± 3.5 ppm, respectively) than before administration (4.0 ± 1.7 ppm).

**Figure 3:**
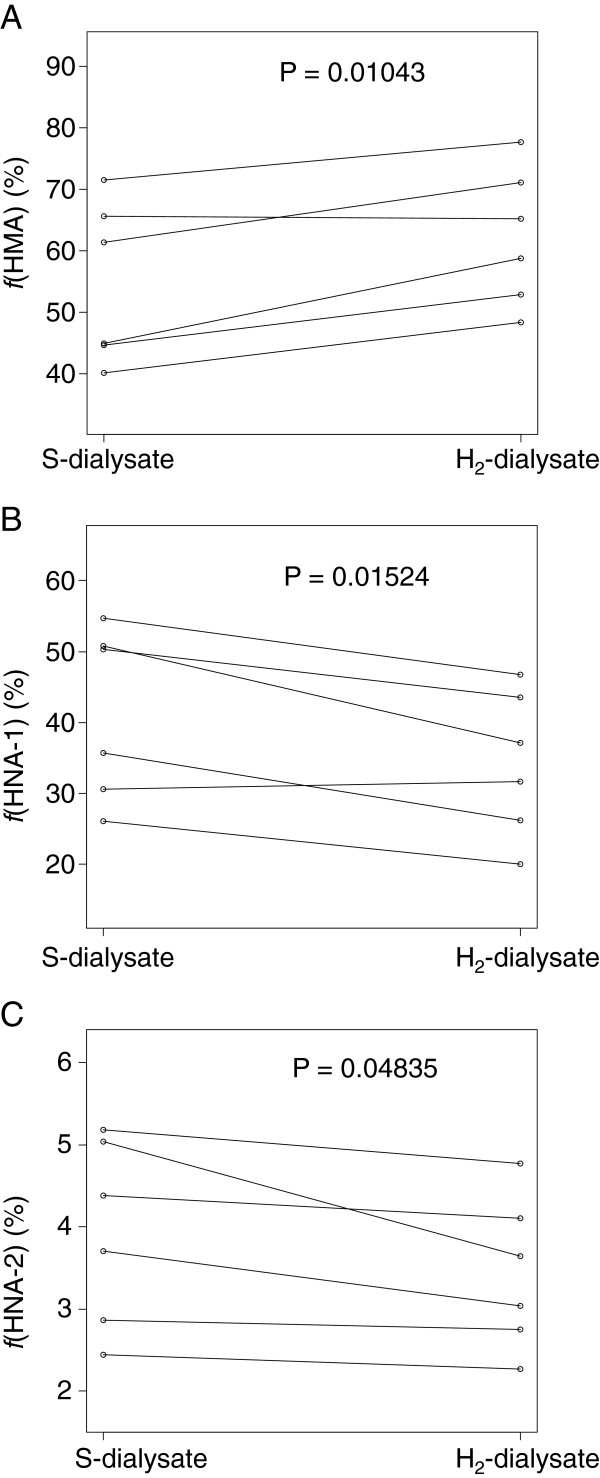
**Redox state of albumin in effluent fluid.** Mean proportion of reduced albumin (ƒ(HMA)) was significantly higher (**A**), and that of oxidized albumin (ƒ(HNA-1) (**B**) and ƒ(HNA-2)) (**C**) was significantly lower in H_2_-enriched dialysate than in standard dialysate.

Figure 3 shows the redox state of albumin in effluent fluid. The mean proportion of HMA (ƒ(HMA)) was significantly higher in H_2_-enriched dialysate (62.31 ± 11.10%) than in standard dialysate (54.70 ± 13.08%). In contrast, ƒ(HNA-1) was significantly lower in H_2_-enriched dialysate (34.26 ± 10.24%) than in standard dialysate (41.36 ± 12.04%). Like ƒ(HNA-1), ƒ(HNA-2) was significantly lower in H_2_-enriched dialysate (3.43 ± 0.92%) than in standard dialysate (3.94 ± 1.13%). These results suggest that the use of H_2_-enriched dialysate reduced peritoneal OS. Regarding the result of fast PET (D/P-Cre, drained volume) and effluent creatinine, albumin, interleukin 6 and carbohydrate antigen 125 levels, no differences were evident between standard and H_2_-enriched dialysate (Table 2).

**Table 2:** The results of serum creatinine value, fast PET and effluent test

	**Standard dialysate**	**H2-enriched dialysate**
Creatinine mg/dL	10.53 ± 2.27	10.03 ± 2.19
Parameter of fast PET		
D/P-Cre	0.71 ± 0.12	0.66 ± 0.11
Drained volume mL/4 hr	470 ± 184	442 ± 130
Effluent test		
Albumin mg/L	408 ± 175	402 ± 145
Interleukin-6 pg/mL	6.0 ± 3.3	5.5 ± 2.3
CA125 U/mL	18.8 ± 8.5	19.5 ± 5.0

**Figure 4:**
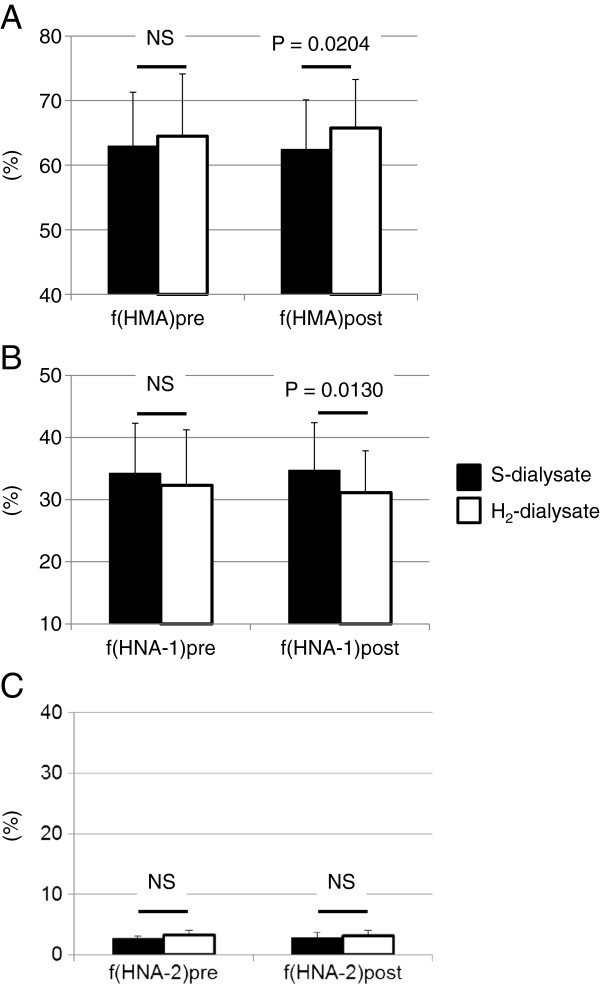
**Redox state of albumin in serum before and after fast PET.** The mean proportion of reduced albumin (ƒ(HMA)) was significantly higher after fast PET using H_2_-enriched dialysate than after that using standard dialysate (**A**). Conversely, the mean proportion of reversibly oxidized albumin (ƒ(HNA-1)) was significantly lower after fast PET using H_2_-enriched dialysate than that after using standard dialysate (**B**). No significant changes were found in irreversibly oxidized albumin (ƒ(HNA-2)) in the both groups (**C**).

Figure 4 shows the redox state of albumin in serum before and after fast PET. The serum ƒ(HMA) level after administration of H_2_-enriched dialysate (65.75 ± 7.52%) was significantly higher than that after standard dialysate (62.44 ± 7.66%). In contrast, ƒ(HNA-1) after administration of H_2_-enriched dialysate (31.12 ± 6.73%) was significantly lower than that of standard dialysate (34.73 ± 7.02%). These results suggest that use of H_2_-enriched dialysate reduced not only peritoneal, but also systemic OS. No significant difference was seen between effluent and serum ƒ(HMA) levels after administration of H_2_-enriched dialysate (65.31 ± 11.10% and 62.71 ± 7.52%, respectively), while effluent ƒ(HMA) after administration of standard dialysate was significantly lower than serum ƒ(HMA) before administration of standard dialysate (54.70 ± 13.08% and 62.96 ± 8.34%, respectively; P = 0.0339), suggesting that intraperitoneal oxidation of albumin was suppressed by H_2_-enriched dialysate.

## Discussion

Several reports have suggested that OS participates in peritoneal deterioration, with findings such as strong cytoplasmic staining of 8-hydroxy-2'-deoxyguanosine in peritoneal biopsy specimens of long-term PD patients [15], amplified protein kinase C signaling and fibronectin expression due to enhanced ROS in cultured human mesothelial cells [16]. In terms of the central role of enhanced OS in PD peritoneal damage, Gunal et al. [17] showed that oral supplementation with the anti-oxidative agent trimetazidine inhibited morphological and functional deterioration of the peritoneum in a PD rat model. However, regarding suppressing OS, no clinical approaches have been available for PD treatment so far.

The present study aimed to test the therapeutic possibility of using dissolved hydrogen in the dialysate to suppress intra-cavity OS in the clinical setting. This study examined the redox state of albumin as a marker of OS. Since the change in redox state of albumin is a physiological and direct reaction, it is appropriate when evaluating real-time OS and/or detecting rapid changes in OS, as compared to other OS markers such as 8-hydroxy-2'--deoxyguanosine, oxidized low-density lipoprotein and F2 isoprotanes, all of which are in vivo by-products during the process of oxidation.

This pilot study of 6 patients clearly demonstrated that single administration of H_2_-enriched dialysate increased levels of both peritoneal and plasma ƒ(HMA) without any detrimental effects.

Intraperitoneal administration of H_2_ altered the local redox state, which may indicate the therapeutic potential of delivering H_2_ directly to the abdominal cavity in respect to the amelioration of peritoneal damage by PD treatment. On the other hand, interestingly, significant increases in serum ƒ(HMA) levels were seen on intraperitoneal administration of H_2_. Rapid changes in hydrogen concentration of expired gas after the administration of H_2_-enriched dialysate may mean that molecular hydrogen in dialysate is rapidly distributed to the body to suppress systemic OS. Another possibility is that increased ƒ(HMA) in the cavity may be recruited into systemic circulation through the abdominal lymphatic drainage. The exact mechanisms underlying increased serum ƒ(HMA) need to be addressed in the future.

In addition, the mechanisms of increased ƒ(HMA) and decreased ƒ(HMA1) by H_2_ have remained unclear in this study. However, molecular hydrogen is known to directly reduce levels of the cytotoxic hydroxyl radical [7], through several possible mechanisms, such as regulation of particular metalloproteins by bonding, or metalloprotein-hydrogen interactions [18]. Whether H_2_ directly reacts with the mercapto-residue of albumin, or H_2_ indirectly modifies it, should be clarified in the future.

Satisfactory anti-oxidative capability of drinking H_2_-enriched water without any detrimental effects has been reported, in both experimental [19-23] and clinical settings, e.g., type II diabetes mellitus [24], metabolic syndrome [25], myopathies (progressive muscular dystrophy and polymyositis/dermatomyositis) [26], and rheumatoid arthritis [27]. In addition, we also reported the clinical feasibility of applying H_2_-enriched water as dialysate for hemodialysis treatment [28,29]. Given these reports and our present findings, H_2_-enriched peritoneal dialysate could be of interest in clinical trials with respect to peritoneal preservation. Furthermore, therapeutic effects seem plausible in terms of the prevention of cardiovascular events in patients, since low f(HMA) has been a significant risk factor for cardiovascular mortality among patients treated with PD [30] and HD [14].

In summary, single administration of H_2_-enriched dialysate reduced peritoneal and systemic OS without any detrimental effects. A longitudinal study is warranted to ensure clinically beneficial effects, such as suppression of peritoneal deterioration and cardiovascular damage.

## Competing interests

The authors declare that they have no competing interests.

## Authors’ contributions

HT, YH, and WJZ carried out the selections of patients, and the sample collections. HT drafted the manuscript. YM, TT, and SE carried out the measurements of samples. SK, and TW contributed to the study as senior advisers. BS carried out the set-up of equipment system for study. MN organized the study project, and drafted the final manuscript. All authors read and approved the final manuscript.
